# Predicting College Success: The Relative Contributions of Five Social/Personality Factors, Five Cognitive/Learning Factors, and SAT Scores

**DOI:** 10.11114/jets.v2i4.451

**Published:** 2014-08-19

**Authors:** Brenda Hannon

**Affiliations:** 1 Department of Psychology and Sociology, Texas A & M University-Kingsville

**Keywords:** GPA, SAT, serf-efficacy, belief of learning, knowledge-integration

## Abstract

To-date, studies have examined simultaneously the relative predictive powers of two or three factors on GPA. The present study examines the relative powers of five social/personality factors, five cognitive/learning factors, and SAT scores to predict freshmen and non-freshmen (sophomores, juniors, seniors) academic success (i.e., GPA). The results revealed many significant predictors of GPA for both freshmen and non-freshmen. However, subsequent regressions showed that only academic self-efficacy, epistemic belief of learning, and high-knowledge integration explained unique variance in GPA (19%-freshmen, 23.2%-non-freshmen). Further for freshmen, SAT scores explained an additional unique 10.6% variance after the influences attributed to these three predictors was removed whereas for non-freshmen, SAT scores failed to explain any additional variance. These results highlight the unique and important contributions of academic self-efficacy, epistemic belief of learning and high-knowledge integration to GPA beyond other previously-identified predictors.

## 1. Introduction

The fast pace of technological and social change over the last 50 years has created a great deal of emphasis on performance in order to achieve one's economic and personal goals. This emphasis is particularly true for college students who are primarily concerned with performing well on measures of academic achievement like overall GPA, an aggregate index of academic performance. *But why do some students excel academically while others do not?* That is, what are the important variables that predict overall GPA?

The present study addresses this question by determining the simultaneous influences of factors from two research areas that are typically separate, namely social/personality and learning/cognition; indeed Credé and Kuncel (2008) label these two content areas as non-cognitive and cognitive. These two content areas were chosen because recent research suggests factors from these areas are highly predictive of performance on the SAT (Hannon & McNaughton-Cassill, 2011), which is a measure of ability that is highly predictive of GPA. Further and as noted below, some researchers argue that models of academic achievement should include both social/personality and cognitive/learning factors in order to be considered comprehensive (e.g., Zeidner, 1998). Thus, the value of the present study is that it not only adds to previous research examining potential predictors of academic achievement but it also reveals the unique and relative contributions of these factors. These findings, in turn, might provide a foundation for a comprehensive theory of GPA specifically and academic achievement generally that includes both social/personality and learning/cognitive factors. Below I briefly describe two models of academic achievement and then describe studies examining just social/personality or just learning/cognitive factors. In the final section, the present study is described.

### 1.1. Background

#### According to Zeidner (1998)

*Any reasonable model of school achievement needs to consider, along with test anxiety, a wide range of cognitive, affective, motivational, somatic, and environmental factors (scholastic abilities, study habits, school attitudes, self-perceptions, and self-efficacy, student health, classroom environment, opportunities for enrichment,* etc.) (pp.259). Likewise, other models of academic achievement also include the social/personality factors of achievement motivation, academic self-efficacy and academic locus of control (e.g., Borkowski, Chan, & Muthukrishna, 2000). Further and consistent with both Zeidner and Borkowski and colleagues, multiple studies have observed correlations between several measures of social/personality and learning/cognitive factors and measures of academic achievement, like exam grades, course grades, semester GPAs and overall GPA (e.g., Chapell et al., 2005; Gore, 2006; Phillips & Gully, 1997; Rose, Hall, Bolen & Webster, 1996; Zimmerman, 1989). However, many of these studies have examined two or perhaps three factors from the same research area simultaneously and very few have examined multiple factors from both social/personality and learning/cognitive research areas. This lack of consideration is quite surprising given Zeider's assertion about the multi-dimensionality of GPA and the fact that many of the correlations between measures of GPA and social/personality and learning/cognitive factors are often modest.

#### 1.1.1 Social/Personality Factors

Multiple studies have investigated the influences of measures of serf-efficacy, test anxiety achievement motivation goals, and locus of control on measures of academic achievement (e.g., Chapell et al., 2005; Elliot & McGregor, 1999; Gore, 2006; Hannon, 2012a; Phillips & Gully, 1997; Robbins et al., 2004; Rose et al., 1996; Zimmerman, 1989). The general findings are that each of these social/personality factors account for significant albeit modest amounts of variance in measures of academic achievement. For example, research suggests that measures of *locus of control* account for about 6.0% of the variance in GPA (e.g., Rose et al., 1996), a finding that presumably occurs because students with higher levels of locus of controls recognize that successful academic outcomes are related to better or more positive personal choices and behaviors rather than negative ones (Borkowski et al, 2000).

Research also suggests that academic achievement is related to *academic self-efficacy,* a construct that includes serf-assessments of one's attainment of academic performance (Bandura, 1986). The logic underlying this research is that positive self-referent beliefs or thoughts are central in positive study habits and behaviors which, in turn, positively stimulate academic performance and motivation (i.e., academic self-efficacy → a student's study behaviors and habits → academic performance and/or motivation). Supporting this theoretical interpretation are studies that demonstrate positive relationships between academic self-efficacy and both motivation and academic performance (e.g., Gore, 2006). In addition, studies show that not only does academic self-efficacy explain as much as 14.3% of the variance in GPA (e.g., Robbins et al., 2004) but it also predicts GPA even when the influences of cognitive ability are controlled (i.e., SAT scores) (Locke, Frederick, Lee & Bobko, 1984).

Students with low self-efficacy also frequently have high *test anxiety*, a “mental state that includes emotional, cognitive, behavioral and bodily reactions” (Hannon & McNaughton-Cassill, 2011, pp. 532; Mcllroy, Bunting & Adamson, 2000). Furthermore, test anxiety is a consistent predictor of GPA such that as test anxiety increases GPA decreases. In a meta-analysis of more than 5400 students, for example, Chapell et al. (2005) demonstrated that test anxiety was indeed inversely related to GPA. Similarly, another meta-analysis reports a −.21 correlation between test anxiety and GPA (e.g., Schwarzer, 1990). From a practical perspective, this correlation suggests that approximately one third of the students (i.e., those with test anxiety) will perform more poorly on exams than the other two thirds of the students (i.e., those with low test anxiety) (Chapell et al., 2005). From a theoretical perspective, the two most common explanations for the inverse test anxiety-GPA relationship are the *interference model* (Mcllroy et al., 2000) and the *deficit model* (Tobias, 1985). According to the *interference model*, test anxiety decreases the efficiencies of cognitive processes which, in turn, decrease academic performance (test anxiety → cognitive processes → academic performance; Mcllroy et al., 2000). In contrast, the *deficit model* suggests that inadequate or deficit learning/test-taking skills have negative direct influences on academic performance (learning/test-taking skills → academic performance; Naveh-Benjamin, McKeachie & Lin, 1987; Tobias, 1985).

Finally, studies suggest that academic performance is also predicted by achievement goals, social/personality factors that reflect the goals or purposes of achievement behavior (Hannon, 2012a; Robbins et al., 2004). *Performance-approach goals* involve demonstrating competence relative to others. Contrastingly, *performance-avoidance goals* involve minimalizing effort. Studies demonstrate that performance-approach goals *positively* influence academic performance whereas performance-avoidance goals *negatively* influence GPA (e.g., Elliot & Church, 1997). Further, *test anxiety* mediates the deleterious influence that performance-avoidance goals exercise on academic performance (e.g., Elliot & McGregor, 1999). Presumably performance-avoidance regulation typically involves avoiding negative outcomes (Elliot and McGregor, 1999). However, in a test taking situation these authors suggest that performance-avoidance regulation probably increases test anxiety, particularly when a test taker focuses on achieving average or normal exam performance while facing possible failure (pp. 629; performance avoidance → test anxiety → academic performance).

#### 1.1.2 Learning/cognitive Factors

With respect to learning/cognitive factors, studies have documented only a few specific learning/cognitive factors that might account for a portion of academic performance (Bridgeman, McCamley-Jenkins & Ervin, 2000; Crede & Kuncel, 2008). For example, a few researchers have observed small positive correlations between semester GPA and measures of fluid intelligence (i.e., *r = .11 to .26;* Coyle & Pillow, 2008) and an indirect relationship between exam grades and epistemic belief of learning, which is operationalized as a belief/awareness regarding of (i) how complex the acquisition of knowledge can be and (ii) the importance of integration during learning (e.g., Muis & Franco, 2009; See Rukavina & Daneman, 1996 for a discussion). However, most studies have assessed the academic performance-cognitive factors relationship by using the SAT because the SAT is assumed to be heavily cognitively loaded (Crede & Kuncel, 2008). Further, a recent meta-analysis suggests that the SAT reliably explains academic performance (e.g., Bridgeman et al., 2000); indeed, SAT scores can explain as much as 13.5% of the variance in GPA (e.g., Robbins et al., 2004).

Yet, using the SAT as the primary measure of cognitive ability is fraught with limitations, especially when one considers that the SAT has been heavily criticized for its lack of construct validity (e.g., Katz, Lautenschlager, Blackburn & Harris, 1990) as well as its inability to be free of biases attributed to socio-economic status (Zwick & Green, 2007).

A recent study by Hannon and McNaughton-Cassill (2011), however, potentially resolves these SAT problems because this study identifies a number of specific factors that predict SAT performance. Ssuch findings are particularly relevant to the present study because they introduce the interesting possibility that the same factors might provide insight into both: (i) the overlapping variance between the SAT and GPA as well as (ii) the unique variance in GPA. More specifically, Hannon and McNaughton-Cassill demonstrated social/personality measures, namely performance-avoidance goals, academic locus of control, and test anxiety, explained 21.4% of the variance in combined SAT scores while learning/cognitive measures (e.g., knowledge integration, working memory, and epistemic belief of learning) explained 37.8% of the variance. Further, when measures for both social/personality and learning/cognitive factors entered into their regression models freely, an even greater proportion of variance in SAT performance was explained. In fact, they observed that both sets of measures explained an impressive 43.3% of the variance in SAT performance (i.e., *r* = .66). With respect to the present study, again these findings are invaluable inasmuch as the specific factors that predict SAT performance might also explain individual differences in GPA. Further, because many of the measures used in Hannon and McNaughton-Cassill’s study assessed factors mentioned in Zeidner’s assertion about the composition of “comprehensive” models of academic achievement (i.e., test anxiety, cognitive factors, scholastic abilities/habits, motivational factors and school attitudes), using the measures selected by Hannon and McNaughton-Cassill allows the present study to assess Zeidner’s assertion about models of academic achievement.

### 1.2 The Present Study

In summary, studies demonstrate that social/personality factors typically explain 6.0 to 14.3% of the variance in GPA and that measures of general cognitive ability, like the SAT, explain about 13% of the variance. However few, if any, studies have examined the simultaneous contributions of multiple social/personality measures, SAT scores, and multiple learning/cognitive measures to GPA. Therefore the present study addresses these shortcomings by using an existing dataset to examine the simultaneous contributions of five social/personality, five learning/cognitive factors, and SAT performance to GPA.

The social/personality measures included: (i) Mcllroy et al.’s (2000) measures of academic self-efficacy and academic locus of control, two relatively short tasks with good Cronbach alphas (i.e., .713+), (ii) three frequently-used and highly reliable measures of test anxiety, namely Benson and El-Zahhar’s (1994), Hodapp and Benson’s (1997), and Sarason’s (1978),, and finally, (iii) Elliot and Church’s (1997) achievement motivation measure, a short commonly-used task with good Cronbach alphas (i.e., .77+). The measures of learning/cognitive factors included: (i) the component processes task (Hannon & Daneman, 2001, 2009), a measure that estimates the cognitive abilities of text memory, text inferencing, knowledge integration, and knowledge access (Cronbach alphas .80+), (ii) the reading span task (Daneman & Carpenter, 1980) and operation span task (Turner & Engle, 1989), and finally, (iii) an epistemic belief of learning questionnaire that has proven to be predictive of cognitive abilities (Hannon & McNaughton-Cassill, 2011).

## 2. Method

### 2.1 Participants

Participants were 348 students who received $40.00 for completing a two-session study that examined the relationships among learning/cognitive abilities, and social-attitudinal beliefs in European-American and Hispanic **students (grant #** SC1 GM081087-03S1). This grant created a large dataset that has results in two published papers and a third paper that is under review. These three papers are substantially different from the present one as none of them examined GPA.

The students included European-American and Hispanic students who were mono-lingual native English speakers and Hispanic students who were dominant English speakers but also spoke some Spanish. The mean age was 19.46, *SD* = 1.72. One hundred and fifty-six students were Hispanic and 192 were European-Americans. Finally, 166 were freshmen and 182 were sophomores, juniors, or seniors.

### 2.2 Measures

Because most of these tasks are commonly used, I describe each measure briefly and then cite references that provide more detailed information.

#### 2.2.1 Cognitive Measures

The component processes task was used to assess *higher-level cognitive processes* (Hannon, 2012b; 2013; 2014 Hannon & Daneman, 2001, 2006, 2009; see Hannon & Frias, 2012 for a preschooler version). Briefly, this task presents relations among two real and three nonsense terms that are described in 3-sentence paragraphs; for example: *A WEMP resembles a WHALE but is larger and weighs more. A whiskered TILN resembles a PIRANHA but is smaller and weighs more. A LORK resembles a TILN but is smaller, weighs more, and is kept as a pet*. Following each paragraph are true-false statements that evaluated four cognitive processes: text memory (e.g., *A WEMP is larger than a WHALE*.), text inferencing (e.g., *A LORK weighs more than a PIRANHA*.), knowledge access (e.g., *A WHALE is larger than a PIRANHA*.), and knowledge integration (e.g., *A WEMP is larger than a PIRANHA*.). Accuracy was the primary dependent variable for each statement type.

Students also responded to 12 items taken from a measure of *epistemic belief of learning* (e.g., Schommer, 1990). A sample item is: *Things are simpler than most teachers would have you believe*. For each item, students indicate their agreement level with a 5-point Likert scale. Smaller/lower scores signify mature knowledge or beliefs about learning whereas larger/higher scores signify naïve or inchoate beliefs about learning.

Finally, students completed two *working memory* measures: the reading span (Daneman & Carpenter, 1980) and the operation span (Turner & Engle, 1989). For each measure, the dependent measure was the total number of words recalled. Scores from the two working memory measures were submitted to two factor analyses—one for freshmen and one for non-freshmen--using promax rotations (i.e., correlated solutions) in order to confirm that both measures assessed the same construct. Both results confirmed that the measures loaded heavily on a single factor; for the freshmen data the eigenvalue of 1.68 explained 83.8% of the variance and for the non-freshmen data the eigenvalue of 1.63 explained 81.5% of the variance. Because both factor analyses resulted in a single factor, a composite score for *working memory* was created by adding the products of each working memory measure’s factor loading with a student’s score for that same measure (i.e., composite working memory = [reading span factor loading × reading span score] + [operation span factor loading × operation span score])

#### 2.2.2 Social/Personality Measures

Students viewed each statement on a computer screen in privacy and then verbally indicated their answer to the research assistant, who entered the response using the keyboard.

The measures of *academic locus of control* and *academic self-efficacy* were 10-item subscales of Mcllroy et al.’s measure (2000). A sample item from the *academic locus of control scale* is: *Thorough reviews before my exams is more than likely to result in a successful outcome*. and a sample item from the self-efficacy scale is: *If I don't understand an academic problem, I persevere until I do*. Each item was accompanied by a 7-point Likert scale. High scores on the academic locus of control measure reflect greater internal locus of control and high scores on the academic self-efficacy measure reflect high self-efficacy.

The three measures of *test anxiety* were: (i) Sarason’s (1978) 37-item true-false test anxiety scale^4^, (ii) Benson and El-Zahhar’s (1994) 20-item revised test anxiety scale and (iii) Hodapp and Benson’s Test 21-item Anxiety Scale (1997). However, because of a computer failure only 36 items of the Sarason scale were used. Scores on these measures were submitted to two factor analyses—one for freshmen and one for non-freshmen—using promax rotations in order to verify that they loaded heavily on the same factor. Both results revealed a single test anxiety factor; for the freshmen data the eigenvalue of 2.50 explained 83.3% of the variance and for the non-freshmen data the eigenvalue of 2.65 explained 88% of the data. Because the three measures loaded on a single factor, a composite score for *test anxiety* was created using the procedure that was used to create the composite measure of working memory.

Finally, students completed the 6-item performance-approach and performance-avoidance scales on an achievement motivation goals measure (i.e., Elliot & Church, 1997). A sample item from the performance-approach scale is: *It is important to me to do better than the other students*; an example item from the performance-avoidance scale is: *I just want to avoid doing poorly in this class*. For each item students select a number from a 7-point Likert scale. High scores suggest a greater propensity to that achievement orientation.

#### 2.2.3 Overall GPA and SAT

GPA and SAT scores were released from university records after students provided written consent. GPA was the average grade points earned at the end of the semester that a student had completed.

## 3. Results

The results consist of three sections. Section one includes data screening and descriptive statistics. Section two reports the correlational and regression analyses for the freshmen data while section three reports the correlational and regression analyses for the non-freshmen data. Significance levels were set to p < .05.

### 3.1 Data Screening and Descriptive Statistics

The data for the freshmen and non-freshmen were screened separately. All data were screened for: (i) skew and kurtosis, (ii) normality (normal probability plots), (iii) linearity (bivariate scatterplots), (iv) data points with too much leverage (hat-values), (v) outliers (*univariate*: studentized residuals, Cook’s D; DFBETAs, and DFITTs; *multivariate statistic*: Mahalanobis distance values), (vi) heteroscedasticity (White’s test), and (vii) multicollinearity (tolerance statistics taken from SAS regression models). Preliminary regression analyses that included all of the measures as predictors were also completed.

As Table 1 shows, the statistics for skewness and kurtosis indicated that all the measures were normal (i.e., all skews and kurtosis < 3.0). Additionally, although a few of the univariate statistics for outliers indicated that a few data points might be problematic, there was no consensus among these statistics for any given data point. In addition multivariate statistic for outliers (i.e., Mahalanobis distance values) indicated there were no problematic data points. Given these findings it was concluded that no data points exerted excessive leverage. In addition, the normal probability plots, bivariate scatterplots, and tolerance tests showed that the data were normal without excessive multicollinearity. Finally, the Mardia’s PKs, a multivariate skew and kurtosis statistic, were well below the recommended 1.96 threshold for both the freshmen and non-freshmen data.

Table 1 depicts the means, standard deviations and ranges for measures. As this table shows, each measure had a large range, which indicatives good variability. In addition, the variabilities are equivalent between the freshmen and non-freshmen, a finding which suggests that any differences in the predictive powers of the measures between the two groups of students cannot be attributed to variability differences.

### 3.2 Freshmen

#### 3.2.1 Correlations

Table 2 reports the correlations for the freshmen. As this table shows, both social/personality (academic self-efficacy, test anxiety and performance-avoidance goals) and learning/cognitive measures (high-knowledge integration, working memory, and epistemic belief of learning) correlated with GPA. Indeed, the sizes of the correlations between the social/personality measures and GPA were the same and, in some instances, greater than the sizes of the correlations between the learning/cognitive measures and GPA, *max r* = .34 versus *max r* = .29. This finding suggests that GPA tends to be more of a consequence of social/personality factors rather than cognitive/learning ones.

When considering all of the correlations in Table 2, academic self-efficacy was the best single social/personality predictor of GPA; it alone explained 11.6% of the variance in GPA (i.e., *r* = .34). On the other hand, the best single learning/cognitive predictor was high-knowledge integration, which explained 8.4% of the variance in GPA (*r* = .29). The fact that a social/personality measure explained more variance in GPA than did a learning/cognitive measure is quite interesting because with SAT scores the predictive powers of these two measures are reversed. That is, the learning/cognitive measure of high-knowledge integration accounts for more variance in performance than does the social/personality measure of academic self-efficacy, 21.2% versus 6.8. These preliminary findings suggest that for freshmen, social/personality factors are more predictive of GPA than are cognitive/learning factors, whereas learning/cognitive factors are more predictive of SAT scores than are social/personality factors.

The other observations worth noting are that the social-personality measures of performance-approach goals and academic locus of control and the learning/cognitive measures of low- and high-knowledge access failed to significantly predict GPA, *r* ≤−*.15*. Thus these four measures were excluded from the regression analyses.

#### 3.2.2 Regression Analyses

Two regression analyses assessed the amount of variance in GPA that was accounted for by the predictors. In the first analysis the social/personality measures entered freely into the model whereas in the second analysis learning/cognitive measures entered freely.

As Table 3 panels (a) and (b) show, the regressions revealed that the social/personality and the learning/cognitive measures each separately explained variance in GPA. Specifically, as Table 3 (a) and 3(b) show, the social/personality measure of academic self-efficacy explained 11.5% of the variance in GPA while the learning/cognitive measures of epistemic belief of learning and high-knowledge integration explained 12.6% of the variance.

Of course, the variance in GPA that was explained when both social/personality and learning/cognitive measures entered freely into a regression model was also of interest. Thus a third regression analysis was completed that allowed the social/personality and learning/cognitive measures to enter simultaneously into the model.

As Table 3(c) reveals, measures from each set of factors explained GPA. Specifically, when both the learning/cognitive and social/personality measures entered a single regression model one social/personality measure, academic self-efficacy, and two learning/cognitive factors, epistemic belief of learning and high-knowledge integration, explained 19.0% of the variance in GPA. This 19.0% variance exceeds both the 11.5% variance explained by just the social/learning measures and the 12.6% variance explained by just the learning/cognitive measures.

In the final two regression analysis I attempted to identify the social/personality and learning/cognitive measures that explain some of the SAT-GPA overlap in variance (i.e., *r* = .49. The first regression entered the social/personality and learning/cognitive measures first and SAT scores second, whereas the second regression entered SAT scores first and the social/personality and learning/cognitive measures second.

As Table 3(d) shows, SAT scores explained an additional 10.6% unique variance in GPA after the academic self-efficacy, high-knowledge integration and epistemic belief of learning measures entered the model. On the other hand as Table 3(e) shows, when SAT scores entered into the model first they explained 24.3% of the variance in GPA and only academic self-efficacy explained a significant proportion of variance. These findings indicate that some of the SAT-GPA overlap in variance is a consequence of all three predictors. In addition, the finding that the social/personality measure of academic self-efficacy explained a significant proportion of variance in GPA is consistent with previous studies that suggest academic self-efficacy explains unique variance in GPA after SAT is controlled (e.g., Locke et al., 1984). Finally, because 24.3% of the variance in GPA is shared with SAT scores but SAT scores explained only 10.6% unique variance after the variance explained by the social/personality and cognitive/learning measures was partialled out, 56.4% of the SAT-GPA shared variance is explained by social/personality and learning/cognitive measures (i.e., 24.3 – 10.6 = 13.7; 13.7/24.3*100 = 56.4%).

### 3.3 Non-Freshmen (i.e., sophomores, juniors, and seniors)

#### 3.3.1 Correlations

Table 4 reports the correlations for the non-freshmen. As Table 4 shows, the SAT-GPA correlation was .31. This .31 is significantly lower than the .49 correlation observed for freshmen, *z* = 1.99, *p* < .03, a finding that indicates that SAT scores are less predictive of GPA as students advance academically. In addition, both the social/personality measures (i.e., academic self-efficacy, academic locus of control, test anxiety and performance-avoidance goals) and learning/cognitive measures (i.e., working memory, the higher-level processes of low-knowledge access, the higher-level process of high-knowledge integration and epistemic belief of learning) explained individual differences in performance on GPA and the sizes of these correlations were not different from those of the freshmen, all *z’ s* < 1.00. Furthermore and also consistent with the freshmen data, the sizes of the correlations between the social/personality measures and GPA were the same as, and in some instances greater than, the sizes of the correlations between the learning/cognitive measures and GPA, *max r* = .41 versus *max r* = −.31. As with the freshmen data, this finding suggests that GPA tends to be more related to social/personality factors rather than cognitive/learning ones.

Also consistent with the freshmen data, academic self-efficacy was the best single social/personality predictor of GPA; academic self-efficacy alone explained 16.4% of the variance in GPA (i.e., *r* = .41). On the other hand and unlike the freshmen data, the best single cognitive/learning predictor was not high-knowledge integration. Rather the best single learning/cognitive predictor was epistemic belief of learning, which explained 9.8% of the GPA variance (i.e., *r* = −31).

In addition and also consistent with the freshmen data, the predictive powers of the factors for GPA were opposite the predictive powers of the factors for SAT scores. That is for SAT performance, epistemic belief of learning explained more variance than did academic self-efficacy, 14.4% versus 6.8% (i.e., *r* = −.38 versus *r* = .26). Finally as depicted in Table 4, performance-approach goals failed to predict GPA, *r* ≤ −.11. For this reason this measure was excluded from the subsequent regression analyses.

#### 3.3.2 Regression Analyses

As with the freshmen data, regressions were completed in order to determine the total proportion of variance in GPA that was explained: (i) by just the social/personality measures, (ii) by just the learning/cognitive measures, and (iii) by both social/personality and learning/cognitive measures. The procedures for these three regressions were the same as those described for the freshmen data.

As Table 5 panels (a) and (b) show, the social/personality measures, academic self-efficacy and performance avoidance goals, explained 20.1% of the variance in GPA while the learning/cognitive measures, epistemic belief of learning and high-knowledge integration, explained 13.3% of the variance. In addition, as Table 5(c) shows, when both social/personality and learning/cognitive measures were considered simultaneously, they explained 23.2% of the variance in GPA. This 23.2% variance is slightly higher than the 19% variance in GPA for freshmen even though the three significant predictors were the same for both groups of students.

Similar to the freshmen data, as a final analysis I completed two additional regression analysis in order to identity the social/personality and learning/cognitive measures that might explain some of the SAT-GPA overlap in variance (i.e., *r* = .31). The procedures for these two regressions were identical to those used with the freshmen data.

As Table 5(d) shows, when SAT scores were entered into the regression model last, they failed to explain any additional unique variance in GPA. On the other hand as Table 5(e) shows, when SAT scores entered first they explained 9.6% of the variance in GPA and the social/personality and learning/cognitive measures explained an additional 13.6% unique variance. More specifically, the social/personality measure academic self-efficacy explained 11.2% variance and the learning/cognitive measure epistemic belief of learning explained 2.4% variance. This latter finding is slightly different from that of the freshmen data inasmuch as for the freshmen data, only academic self-efficacy explained unique variance in GPA. Finally, because 9.6% of the variance in GPA is shared with SAT scores, but SAT scores failed to explain any unique variance once the variance attributed to the social/personality and learning/cognitive measures was removed, it can be concluded that 100% of the shared variance between SAT scores and GPA for non-freshmen is explained by social/personality and learning/cognitive measures.

## 4 Discussion

Although it is generally accepted that a number of factors contribute to performance on measures of academic achievement (e.g., course grades, semester GPA, overall GPA), few studies have considered more than two or three factors simultaneously. This shortfall is quite surprising given that many of these factors have modest correlations with GPA and multiple researchers have recommended that models of school achievement should consider multiple social/personality and learning/cognitive factors (Zeidner, 1998). The present study begins to address these shortcomings by comparing the relative contributions of five social/personality factors and five learning/cognitive factors to GPA.

With respect to the influences of just social/personality factors and just learning/cognitive factors, the results were fairly consistent between the freshmen and non-freshmen (i.e., sophomores, juniors, seniors). For both groups of students, academic self-efficacy was the best social/personality predictor, explaining 11.5 to 16.4% of the total variance in GPA and epistemic belief of learning and high-knowledge integration were the best cognitive/learning predictors, explaining 12.6 to 13.3% of the total variance in GPA. Further, when social/personality and learning/cognitive factors were considered simultaneously, three measures—academic serf-efficacy, epistemic belief of learning, and high-knowledge integration—explained an even greater proportion of variance in GPA, for freshmen they explained 19% of the total variance while for non-freshmen they explained 23.2%.

Of course, skeptics might argue that showing that social/personality and learning/cognitive measures predict GPA is not especially surprising. After all, many studies have demonstrated that these measures academic achievement. I am the first to agree that these individual correlations are not particularly novel. However, construing the present results as not useful or informative is the same as misconstruing the importance of students’ and educators’ need to understand which measures are most important for improving GPA. After all, the present study did test 10 social/personality and cognitive/learning measures; yet it showed that only three of these measures uniquely contributed to GPA.

The present findings also inform a number of previous assertions about models of academic achievement. Zeidner (1998), for instance, asserts that the composition of “comprehensive” models of academic achievement should include measures of both social/personality factors and cognitive/learning factors. Similarly, Crede and Kuncel (2008) argue that study attitudes, skills, and habits are all predictors of academic performance. Consistent with both of these assertions, the present study observed that many of the social/personality and learning/cognitive measures correlated with GPA. However, the present study also showed that although many of these measures correlate with GPA, only three social/personality and learning/cognitive measures explained unique variance in GPA.

Additionally, consistent with Borkowski et al., (2000) the present study observed that achievement, academic serf-efficacy, and locus of control all predicted GPA. The present results however go beyond those of Borkowski and colleagues by showing that learning/cognitive factors, like epistemic belief of learning and high-knowledge integration, also predict GPA.

The present study also examined whether social/personality and learning/cognitive factors might explain some of the overlapping variance between the SAT and GPA. The regression analyses revealed that 56.4 to 100% of this shared variance was explained by social/personality and learning/cognitive measures. Further, because the proportion of variance explained by three predictors (academic self-efficacy, epistemic belief, and high-knowledge integration) decreased when SAT scores entered into the model first, it can be concluded that most of the variance shared between SAT scores and GPA is a result of these three predictors.

The results of the SAT-GPA analyses also revealed some minor differences between the data for the freshmen versus the non-freshmen. For freshmen, SAT scores explained significantly more variance in GPA than they did for non-freshmen, i.e., *r* = .49 versus *r* = .31. This finding suggests that although SAT scores are highly predictive of freshmen GPA, as time passes and students become sophomores, juniors and seniors, SAT scores become less and less predictive of academic achievement. This interpretation is further supported by the regression analyses which showed that for freshmen, the SAT explained an additional 10.6% unique variance in GPA once the variance for the social/personality and learning/cognitive predictors was partialled out whereas for non-freshmen, SAT scores failed to explain any additional significant variance in GPA.

As noted in the results section, the present study also revealed an interesting difference between SAT and GPA. Specifically, whereas SAT performance tends to have stronger correlations with measures of learning/cognitive factors as opposed to social/personality factors, GPA tended to have stronger correlations with measures of social/personality factors than learning/cognitive factors. This finding suggests that in order to improve GPA, students and educators should focus more on interventions that improve social/personality factors rather than learning/cognitive ones. On the other hand, in order to improve SAT performance students and educators should focus more on interventions that improve learning/cognitive factors rather than social/personality factors. Furthermore this finding also suggests that theoretical models designed to explain SAT performance will be different from theoretical models designed to explain GPA.

Of course, there are limitations in the present study. One obvious limitation is that the students attended the same university rather than multiple universities. A second limitation is that only a single measure for most of the predictors rather than multiple ones. Consequently it is possible that the results are restricted to just the measures administered in the present study. A third limitation is that the present study does not establish causality. After all, *GPA* was collected *after* the measures in the present study were administered whereas *SAT performance* was assessed *before*.

Finally, although the present study determined the unique and relative contributions of a number of social/personality and learning/cognitive factors to GPA, it did not develop or test a specific model of academic achievement. Nevertheless the present study does provide an important empirical foundation for such research because it shows which social/personality and learning/cognitive factors have the most influence on GPA (i.e., academic self-efficacy, epistemic belief of learning, and high-knowledge integration) and those factors that have a lesser influence (i.e., academic locus of control, test anxiety, performance avoidance, and working memory). Using these findings as a starting point, future research can test theoretical models such as Phillips and Gully’s model (1997) that suggests that cognitive ability and locus of control influence self-efficacy which, in turn, influences academic performance (i.e., cognitive ability, locus of control → self-efficacy → academic performance). Or, it can be used to compare Phillip and Gully’s model to that of Elliot and McGregor (1999), a model that suggests performance-avoidance goals influence test anxiety and test anxiety influences exam performance (i.e., performance-avoidance goals → test anxiety → exam performance).

In conclusion, no study has examined simultaneously the social/personality factors of academic locus of control, academic self-efficacy, test anxiety, and achievement goals within the same study, nor have the influences of these factors been examined in concert with measures of cognitive/learning factors. The present study eliminates these shortcomings and reveals that the three major contributors to academic success are academic self-efficacy, epistemic belief of learning, and high-knowledge integration. Indeed, the variance in GPA that is explained by these three measures is greater than the variance explained in many of the studies reviewed in the introduction. These results should, in turn, inform educators as to which factors are most important when considering increasing academic success.

## Figures and Tables

**Table 1:** Descriptive Statistics for GPA, SAT and Measures completed for Freshmen and Non-freshmen (i.e., Sophomores, Juniors, and Seniors).

	Freshmen (*n* = 166)			Sophomores, Juniors, Seniors (*n* = 182)		
Measure	M (SD)	Range	Skew(Kurt)	M (SD)	Range	Skew(Kurt)
Measures of academic achievement						
SAT (max = 1600)	1064.00 (152.01)	690 – 1540	−.44(−.57)	1059.00 (151.87)	710 – 1450	.11(−.26)
GPA (max = 4.0)	2.85 (0.85)	0.00 – 4.0	−.87(.66)	2.95 (0.67)	0.60 – 4.0	−.49(.02)
Measures of higher-level processes						
High-knowledge integration (max = 36)	26.77 (5.54)	13 – 36	−.43(−.57)	26.97 (5.04)	15 – 36	−.33(−.79)
Low-knowledge access (max = 36)	33.49 (2.07)	25 – 36	−1.22(1.95)	33.57 (2.24)	24 – 36	−1.59(2.34)
High-knowledge access (max = 24)	22.18 (1.99)	15 – 24	−1.44(1.96)	22.45 (1.51)	17 – 24	−1.16(1.14)
Measures of working memory						
Reading span (max = 100)	57.02 (10.86)	36 – 81	.21(−.83)	56.60 (10.95)	32 – 85	.40(−.08)
Operations span (max = 100)	72.22 (13.08)	46 – 98	−.11(−.77)	72.77 (11.85)	37 – 99	−.14(−.44)
Composite working memory	117.50 (19.93)	75 – 159	.07(.81)	117.56 (18.71)	66 – 164	.18 (−.29)
Epistemic belief of learning (max = 60)	35.21 (4.46)	24 – 45	−.06(−.65)	35.04 (5.22)	20 – 47	−.18(−.06)
Academic self-efficacy (max = 70)	51.48 (8.33)	30 – 69	−.34(−.33)	52.31(7.63)	29 – 69	−.34(−.09)
Academic locus of control (max = 70)	53.25 (7.50)	30 – 70	−.45(.24)	54.76 (7.17)	34 – 70	−.58(.15)
Test anxiety	93.72 (22.14)	55 – 155	.53(−.21)	96.44(25.38)	54 – 170	.74(.07)
Performance-approach goals (max = 7)	5.02 (1.26)	1 – 7	−.66(.03)	5.02(1.37)	1 – 7	−.54(−.02)
Performance-avoidance goals (max = 7)	4.55 (1.11)	1 – 7	−.43(−.24)	4.57(1.19)	1 – 7	−.68(−.25)

**Table 2:** Correlations among SAT, GPA, Measures of Higher-level Processes, Working Memory, Epistemic Belief, Academic Self-efficacy, Academic Locus of Control, Test Anxiety, Performance-approach goals, and Performance-avoidance goals for Freshmen (*n =166*).

		1	2	3	4	5	6	7	8	9	10	11	12
1.	SAT	---	.49*	.46*	.26*	.25*	.45*	−.38*	.26*	.14	−.37*	.07	−.33*
2.	GPA		---	.29*	.15	.07	.23*	−.27	.34*	.12	−.25*	.12	−.20*
3.	High-know integration			---	.37*	.30*	.34*	−.23	.23*	.24*	−.31*	−.08	−.30*
4.	low-knowledge access				---	.45*	.20*	−.20	.09	.22*	−.24*	.02	−.22*
5.	High-knowledge access					---	.16*	−.09	.12	.14	−.30*	.02	−.13
6.	Composite working memory						---	−.28	.14	.10	−.22*	.07	−.13
7.	Epistemic Belief of Learning							---	−.18*	−.03	.18*	.03	.19*
8.	Academic self-efficacy								---	.59*	−.53*	.17*	−.26*
9.	Academic locus of control									---	−.41*	.13	−.20*
10.	Test Anxiety										---	.13	.60*
11.	Performance-approach goals											---	.20*
12.	Performance-avoidance goals												---

Note.

* p < .05; for *r* < .151, *p* > .05.

**Table 3:** Regression Analyses of GPA using Measures of Social/Personality Factors, Cognitive/Learning Factors and GPA as Predictors for Freshmen (*n* = 166).

	Variable	*R*	*R*^2^	Δ*R*^2^	*F*
	(a) Social/Personality predictors of GPA				
1.	Academic self-efficacy	.339	.115	.115	21.21*
2.	Performance avoidance	.358	.128	.013	2.46^a^
3.	Academic locus of control	.374	.140	.012	2.33^a^
	(b) Cognitive/Learning predictors of GPA				
1.	High-knowledge integration	.286	.082	.082	14.60*
2.	Epistemic belief of learning	.355	.126	.044	8.19*
	(c) Social/Personality and Cognitive/Learning predictors of GPA				
1.	Self-efficacy	.339	.115	.115	21.21*
2.	High-knowledge integration	.400	.160	.045	8.82*
3.	Epistemic belief of learning	.436	.190	.030	6.05*
	(d) SAT as last predictor of GPA				
1.	Self-efficacy	.339	.115	.115	21.21*
2.	High-knowledge integration	.400	.160	.045	8.82*
3.	Epistemic belief of learning	.436	.190	.030	6.05*
		
4.	SAT	.544	.296	.106	24.22*
	(e) GPA - SAT as first predictor				
1.	SAT	.493	.243	.243	52.49*
		
2.	Self-efficacy	.539	.290	.047	10.85*
3.	High-knowledge integration	.539	.291	.001	0.36
4.	Epistemic belief of learning	.544	.296	.005	1.10

Note.

* *p* < *.05*;

a *p* < *.15*.

**Table 4:** Correlations among SAT, GPA, Measures of Higher-level Processes, Working Memory, Epistemic Belief, Academic Self-efficacy, Academic Locus of Control, Test Anxiety, Performance-approach goals, and Performance-avoidance goals for Non-freshmen *(n = 182)*.

		1	2	3	4	5	6	7	8	9	10	11	12
1.	SAT	---	.31*	.41*	.24*	.24*	.43*	−.38*	.26*	.07	−.34*	−.10	−.34*
2.	GPA		---	.26*	.20*	.08	.16*	−.31*	.41*	.25*	−.26*	.11	−.31*
3.	High-know integration			---	.40*	.28*	.31*	−.25*	.18*	.10	−.26*	−.09	−.17*
4.	Low-knowledge access				---	.38*	.14	−.15*	.22*	.07	−.29*	−.03	−.12
5.	High-knowledge access					---	.08	−.10	.05	.04	−.07	−.05	−.15*
**6.**	Composite working memory						---	−.31*	.11	.07	−.14	−.14	−.23*
7.	Epistemic Belief of Learning							---	.26*	−.01	.31*	.17*	.39*
8.	Academic self-efficacy								---	.46*	−.52*	.17*	−.32*
9.	Academic locus of control									---	−.34*	.04	−.15*
10.	Test Anxiety										---	.25*	.57*
11.	Performance-approach goals											---	.26*
12.	Performance-avoidance goals												---

*Note*.

* *p* < .05; for *r* < .15, *p* > .05.

**Table 5:** Regression Analyses of GPA using Measures of Social/Personality Factors, Cognitive/Learning Factors and GPA as Predictors for Non-freshmen (*n* = 182).

	Variable	*R*	*R^2^*	Δ*R^2^*	*F*
(a) Social/Personality Predictors of GPA					
1.	Academic self-efficacy	.405	.164	.164	35.39*
**2.**	Performance avoidance	.448	.201	.037	8.21*
**3.**	Performance approach	.462	.213	.012	2.73^a^
(b) Cognitive/Learning Predictors of GPA					
**1.**	Epistemic belief of learning	.313	.098	.098	19.49*
2.	High-knowledge integration	.365	.133	.035	7.22*
(c) Social/Personality and Cognitive/Learning Predictors of GPA					
1.	Self-efficacy	.405	.164	.164	35.39*
2.	Epistemic belief of learning	.459	.211	.047	10.62*
**3.**	High-knowledge integration	.482	.232	.021	4.95*
4.	Performance avoidance	.497	.247	.015	3.32^a^
(d) SAT as last predictor of GPA					
1.	Self-efficacy	.405	.164	.164	35.39*
2.	Epistemic belief of learning	.459	.211	.047	10.62*
**3.**	High-knowledge integration	.482	.232	.021	4.95*
			
4.	SAT	.493	.243	.011	2.39^a^
(e) SAT as first predictor of GPA					
1.	SAT	.310	.096	.096	18.92*
			
2.	Self-efficacy	.456	.208	.112	25.50*
3.	Epistemic belief of learning	.482	.232	.024	5.54*
4.	High-knowledge integration	.493	.243	.011	2.52^a^

*Note*.

* p < .05;

a p < .15.
